# Murine Typhus, Algeria

**DOI:** 10.3201/eid1404.071376

**Published:** 2008-04

**Authors:** Nadjet Mouffok, Philippe Parola, Didier Raoult

**Affiliations:** * Service des Maladies Infectieuses CHU’Oran, Oran, Algeria; † World Health Organization Collaborative Centre for Rickettsial and Arthropod-borne Bacterial Diseases, Marseilles, France

**Keywords:** Rickettsia, Algeria, Africa, typhus, rickettsiosis, fleas, letter

**To the Editor:** Rickettsioses, or typhoid diseases, are caused by obligate intracellular bacteria of the order Rickettsiales. The ubiquitous murine typhus is caused by *Rickettsia typhi*. Although cat fleas and opposums have been suggested as vectors in some places in the United States, the main vector of murine typhus is the rat flea (*Xenopspylla cheopis),* which maintains *R. typhi* in rodent reservoirs (*1*). Most persons become infected when flea feces containing *R. typhi* contaminate broken skin or are inhaled, although infections may also result from flea bites (*1*). Murine typhus is often unrecognized in Africa; however, from northern Africa, 7 cases in Tunisia were documented in 2005 (*2*).

We conducted a prospective studyin Algeria which included all patients who had clinical signs leading to suspicion of rickettsioses (high fever, skin rash, headache, myalgia, arthralgia, eschar, or reported contact with ticks, fleas, or lice) who visited the Oran Teaching Hospital in 2004–2005 for an infectious diseases consultation. Clinical and epidemiologic data as well as acute-phase (day of admission) and convalescent-phase (2–4 weeks later) serum samples were collected. Serum samples were sent to the World Health Organization Collaborative Center for Rickettsial Diseases in Marseille, France. They were tested by immunofluoresence assay (IFA), by using spotted fever group (SFG) rickettsial antigens (*R. conorii conorii*, *R. conorii israelensis*, *R. sibirica mongolitimonae*, *R. aeschlimmanii*, *R. massiliae*, *R. helvetica*, *R. slovaca*, and *R. felis*) and *R. typhi* and *R. prowazekii* as previously reported (*3*). When cross-reactions were noted between several rickettsial antigens, Western blot (WB) assays and cross-absorption studies were performed as previously described (*4*). A total of 277 patients were included. We report 2 confirmed cases of *R. typhi* infection in patients from Algeria.

The first patient, a 42-year-old male pharmacist who reported contact with cats and dogs parasitized by ticks, consulted with our clinic for a 10-day history of high fever, sweating, headache, arthralgia, myalgia, cough, and a 6-kg weight loss. He had not received any antimicrobial drugs before admission. No rash, eschar, or specific signs were found. Standard laboratory findings were within normal limits. No acute-phase serum sample was sent for testing. However, IFAs on convalescent-phase serum were negative for SFG antigens (except *R. felis*: immunoglobulin [Ig] G 64, IgM 128), but they showed raised antibodies against *R. typhi* and *R. prowazekii* (IgG 2,048, IgM 1,024).

The second patient, a 25-year-old farmer, was hospitalized for a 5-day history of fever, headache, diarrhea, and lack of response to treatment with amoxicillin and acetaminophen. He reported contact with cats and cattle. A discrete macular rash and pharyngitis were observed. Standard laboratory findings were within normal limits, except neutrophil count was elevated at 11.2/μL (normal levels 3–7/μL). Acute-phase serum was negative for rickettsial antigens. Convalescent-phase serum obtained 2 weeks later was positive for several SFG antigens (IgM only; the highest level was 256 for *R. conorii*), and higher levels of antibodies were obtained against *R. typhi* and *R. prowazekii* (IgG 256, IgM 256). WB and cross-absorption studies confirmed *R. typhi* infection (Figure). Both patients recovered after a 3-day oral doxycycline regimen and have remained well. (A single 200-mg dose of oral doxycycline usually leads to defervescence within 48–72 hours [*1*]).

**Figure:**
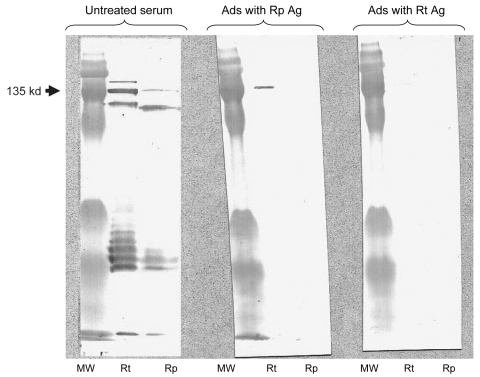
Western blot assay and cross-adsorption studies of an immunofluoresence assay–positive serum sample from a patient with rickettsiosis in Algeria. Antibodies were detected at the highest titer (immunoglobulin [Ig] G 256, IgM 256) for both *Rickettsia typhi* and *R. prowazekii* antigens. Columns Rp and Rt, Western blots using *R. prowazekii* and *R. typhi* antigens, respectively. MW, molecular weight, indicated on the left. When adsorption is performed with *R. typhi* antigens (column Ads with Rt Ag), it results in the disappearance of the signal from homologous and heterologous antibodies, but when it is performed with *R. prowazekii* antigens (column Ads with Rp Ag), only homologous antibody signals disappear, indicating that the antibodies are specific for *R. typhi*.

Murine typhus is a mild disease with nonspecific signs. Less than half of patients report exposure to fleas or flea hosts. Diagnosis may be missed because the rash, the hallmark for rickettsial diseases, is present in <50% of patients and is often transient or difficult to observe. Arthralgia, myalgia, or respiratory and gastrointestinal symptoms, as reported here, are frequent; neurologic signs may also occur (*5*). As a consequence, the clinical picture can mimic other diseases. A review has reported 22 different diagnoses that were proposed for 80 patients with murine typhus in the United States (*6*).

Serologic tests are the most frequently used and widely available methods for diagnosis of rickettsioses (*7*). IFA is the reference method (*7*). However, *R. typhi* may cross-react with other rickettsial antigens, including SFG rickettsiae, but especially with the other typhus group rickettsia, *R. prowazekii*, the agent of epidemic typhus (*8*). Epidemic typhus is transmitted by body lice and occurs more frequently in cool areas, where clothes are infrequently changed, and particularly during human conflicts. It is still prevalent in Algeria (*9*).

This cross-reactivity led to some difficulties in interpreting serologic results (*10*). However, WB and cross-adsorption studies can be used when cross-reactions occur between rickettsial antigens. They are useful for identifying the infecting rickettsia to the species level and for providing new data about the emergence or reemergence of rickettsioses, as reported here. These assays are, however, time-consuming and only available in specialized reference laboratories.

Clinicians need to be aware of the presence murine typhus in Algeria, especially among patients with unspecific signs and fever of unknown origin. Tetracyclines remain the treatment of choice.
